# Dugas’ Bandage for the Breast

**Published:** 1885-04-20

**Authors:** T. L. Lallerstedt

**Affiliations:** Georgia


### DUGAS' BANDAGE FOR THE BREAST.

By T. L. Lallerstedt, M. D., of Georgia.

I see, on page 355 of "Transactions of the Georgia Medical Association," a new mammary bandage by W. B. Park, M. D., and in Vol. XVIII, No. 1, of the American Journal of Obstetrics, "Treatment of Mastitis by Bandaging and Rest." From reading Dr. Park's article, I notice that his bandage has objections, because it is applied with some trouble. Dr. Harris' plan, as in his second article, is a very complicated one. The late Prof. L. A. Dugas always told his classes to use the many-tailed bandage for any soreness of the breast of females. Take a piece of cloth long enough to go around the patient's body, and cut out a hole in it large enough to admit the sound breast, so as to make no pressure on it. For the affected breast, cut a hole just large enough for the nipple. Put it over the chemise, if you like, or a female attendant can apply it. As the swelling decreases, any one can tighten the bandage. Where ladies desire to wean their children, using this bandage soon dries up the milk without pain.

This bandage is so simple and easy of application any woman can make and put it on. I have used it for about seventeen years, and never have had to open a rising breast in my practice yet.

The bandage should be long enough to go around the body and wide enough to cover the entire breast—say six or eight inches—the two ends being slit into strips about an inch wide, constituting a many-tailed bandage, which may be made to adhere so as give uniform pressure upon the breast. This may be done by pinning the ends together, and tightening from time to time if it gets slack.

